# The circular RNA NT5E promotes non-small cell lung cancer cell growth via sponging microRNA-134

**DOI:** 10.18632/aging.102861

**Published:** 2020-02-25

**Authors:** Lingyun Dong, Jiangnan Zheng, Yun Gao, Xiaoting Zhou, Weizhen Song, Jianan Huang

**Affiliations:** 1 Department of Respiratory Medicine, The First Affiliated Hospital of Soochow University, Suzhou, China; 2 Department of Respiratory Medicine, Affiliated Wujiang Hospital of Nantong University, Suzhou, China; 3 Department of General Surgery, The Third Affiliated Hospital of Soochow University, Changzhou, China

**Keywords:** circNT5E, miR-134, NSCLC, cancer progression

## Abstract

The current study tested expression and potential function of circular RNA ecto-5’-nucleotidase (circNT5E) in human non-small cell lung cancer (NSCLC). We show that circNT5E levels are significantly elevated in human NSCLC tissues and cells, correlating with downregulation of its potential targets, miR-134, miR-422a and miR-338. In A549 and primary NSCLC cells, circNT5E shRNA inhibited cancer cell growth, proliferation and migration, whiling inducing apoptosis activation. Conversely, ectopic circNT5E overexpression promoted A549 cell progression *in vitro*. miR-134 is the primary target of circNT5E in lung cancer cells. RNA-Pull down assay in A549 cells confirmed the direct association between biotinylated-miR-134 and circNT6E. miR-134 levels were significantly increased in circNT5E-silenced A549 cells, but reduced with circNT5E overexpression. Forced overexpression of miR-134 mimicked circNT5E shRNA-induced actions, inhibiting NSCLC cell growth and proliferation. In contrast, miR-134 inhibition largely attenuated circNT5E shRNA-induced anti-NSCLC cell activity. Importantly, circNT5E shRNA was ineffective in miR-134-overexpressed A549 cells. Collectively, circNT5E promotes human NSCLC cell progression possibly by sponging miR-134.

## INTRODUCTION

Lung cancer is a global health threat [1, 2]. Despite recent improvements in the early diagnosis and newly-developed therapies, the five-year overall survival for lung cancer patients is still low (15%) [3–5]. Further, the lung cancer’s incidence is rising, particularly in Eastern countries [6–8]. Non-small cell lung cancer (NSCLC) accounts for over 80% of all lung cancers [1, 3]. The poor prognosis is partially due to the limited understanding of NSCLC biology [3–5]. It is therefore urgent to further explore the pathological mechanisms for NSCLC progression [3–5].

Non-coding RNAs (ncRNAs) include microRNA (miRNAs), long non-coding RNAs (LncRNAs) and circular RNAs (circRNAs) [9, 10]. Recent studies have proposed the pivotal roles of the ncRNAs in tumorigenesis and progression of NSCLC and other lung cancers [11]. Unlike other ncRNAs, circRNAs can form highly stable circular structure via joining of the 3′ and 5′ terminals [10, 12, 13]. circRNAs function as miRNAs sponges, altering gene expression and cancerous behaviors in tumor cells [10, 13, 14].

Dysregulation of circRNAs is often detected in NSCLC [15–18]. For example, Wang et al., showed that the circRNA circ_0067934 overexpression correlates with unfavorable prognosis in NSCLC. Silencing of circ_0067934 inhibited proliferation and migration of NSCLC cells [19]. Furthermore, the circRNA circ_0016760 upregulation indicates unfavorable prognosis in NSCLC [20, 21]. Similarly, circular RNA hsa_circ_0007534 upregulation predicts poor prognosis in NSCLC [22]. On the contrary, Lie et al., reported that the circular RNA circ_0001649 inhibited NSCLC cell progression via sponging miR-331-3p and miR-338-5p [23].

Wang et al., have discovered one circRNA derived from ecto-5′-nucleotidase (NT5E), or circNT5E [24]. It is overexpressed in human glioblastoma [24]. circNT5E sponges microRNA-422a (miR-422a) and possible other tumor-suppressive miRNAs to promote glioblastoma cell growth [24]. Conversely, circNT5E silencing potently inhibited glioblastoma cell progression *in vitro* and *in vivo* [24]. The expression and potential functions of circNT5E in human NSCLC cells are tested here.

## RESULTS

### circNT5E is upregulated in human NSCLC tissues and cells

**Figure 1:**
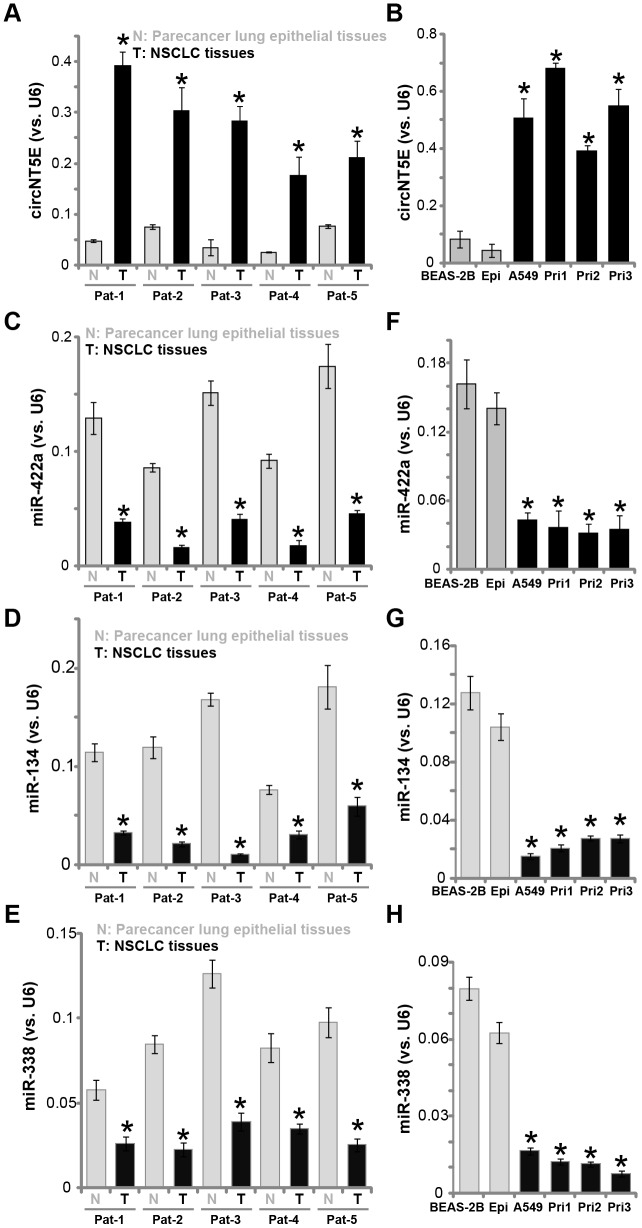
**circNT5E is upregulated in human NSCLC tissues and cells.** Total RNA was extracted from the described human tissues and cells, expression of circNT5E (**A** and **B**), miR-422a, miR-134 and miR-338 (**C**–**H**) was tested by qPCR, with results normalized to *U6 RNA*. “Pat” stands for NSCLC patient number. Each tumor or epithelial tissue was randomly cut into five pieces, with expression of listed genes tested. Error bars stand for mean ± standard deviation (SD, n=5). * *P* < 0.05 vs. lung epithelial tissues (“N”)/cells (“Epi”). Experiments in this figure were repeated five times, and similar results were obtained.

First, circNT5E expression in human NSCLC tissues was examined. A total of five pairs of NSCLC tissues (“T”) and paracancer lung epithelial tissues (“N”) were obtained. qPCR assays were performed to test circNT5E expression. Results in Figure 1A demonstrated that circNT5E is significantly elevated in all tested cancer (“T”) tissues, as compared its expression in the normal epithelial tissues. Furthermore, circNT5E expression is elevated in A549 and primary human NSCLC cells (“Pri-1/-2/-3” [25]) (Figure 1B). Its expression is however relatively low in BEAS-2B lung epithelial cells [26–27] and in the primary human lung epithelial cells (“Epi” [25]) (Figure 1B).

It has been previously shown that circNT5E functions as the sponges of multiple tumor-suppressive miRNAs, including miR-422a, miR-134 and miR-338 [24, 28–31]. We therefore tested the expression of these miRNAs in NSCLC tissues and cells. As demonstrated, levels of miR-422a, miR-134 and miR-338 are all decreased in the NSCLC tissues (Figure 1C–1E), as well as in the established and primary NSCLC cells (Figure 1F–1H). In contrary, miR-422a, miR-134 and miR-338 expression is relatively high in lung epithelial tissues (Figure 1C–1E) and epithelial cells (Figure 1F–1H). These results show that circNT5E is upregulated in NSCLC tissues and cells, correlating with downregulation of its targets, miR-422a, miR-134 and miR-338.

### circNT5E silencing inhibits NSCLC cell growth, proliferation and migration

**Figure 2:**
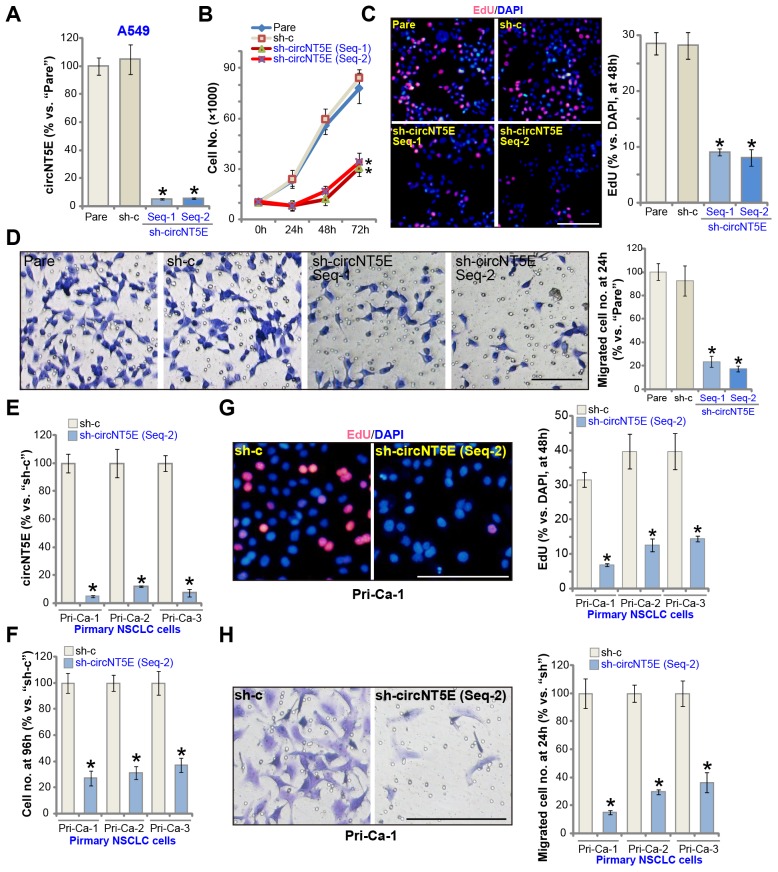
**circNT5E silencing inhibits NSCLC cell growth, proliferation and migration.** The stable A549 cells (**A**–**D**) or the primary human NSCLC cells (Pri-Ca-1/-2/-3, E-H) with the lentivirus-packaged circNT5E shRNA (“sh-circNT5E-Seq-1/2”, two different sequences) or the non-sense control shRNA (“sh-c”) were cultured, the circNT5E expression was tested by qPCR (**A** and **E**), cell growth (cell counting assay, **B** and **F**), proliferation (EdU incorporation, **C** and **G**) and migration (“Transwell” assay, **D** and **H**) were tested by the mentioned assays; “Pare” stands for the parental control cells (Same for all Figures). Error bars stand for mean ± standard deviation (SD, n=5). * *P* <0.05 vs. “Pare”/“sh-c” cells. Experiments in this figure were repeated five times, and similar results were obtained. Bar= 100 μm (**C**, **D**, **G** and **H**). For all the functional assays exact same number of viable NSCLC cells with applied genetic treatments were initially seeded into each well/dish (at 0h), and cells cultured for applied time periods (Same for all Figures).

FISH assay results demonstrated that circNT5E mainly localized in the cytoplasm of A549 cells (Supplementary Figure 1A). To examine the potential activity of circNT5E on the functions of human NSCLC cells, two lentiviral constructs with shRNA targeting non-overlapping sequence of circNT5E, sh-circNT5E-Seq-1 and sh-circNT5E-Seq-2, were established. The two were individually transduced to A549 cells. Followed by puromycin selection the stable cell lines were established. Analyzing circNT5E expression, by qPCR, confirmed that the applied sh-circNT5E resulted in over 90% decrease of circNT5E expression in the stable cells (*vs.* parental control cells, Figure 2A). circNT5E shRNA did not alter the expression of NT5E protein, which was encoded by the linear *NT5E mRNA* (Supplementary Figure 1B). Significantly, cell counting assay results demonstrated that circNT5E shRNA significantly inhibited A549 cell growth (Figure 2B). A549 cell proliferation, tested by the BrdU incorporation assay, was largely inhibited as well in sh-circNT5E-expressing A549 cells (Figure 2C). Furthermore, “Transwell” assay results, Figure 2D, demonstrated that circNT5E silencing led to significant suppression on A549 cell migration. The scramble control shRNA, sh-c, did not alter circNT5E expression (Figure 2A) and A549 cell functions (Figure 2B–2D).

The primary human NSCLC cells, derived from three independent patients, Pri-Ca-1/-2/-3, were cultured and transduced with sh-circNT5E-Seq-2. The latter induced over 90% inhibition of circNT5E expression in primary cancer cells (Figure 2E). The cell counting assay results, Figure 2F, demonstrated that circNT5E shRNA potently inhibited growth of the primary NSCLC cells. Additionally, the primary cancer cells with sh-circNT5E-Seq-2 showed significantly inhibited cell proliferation (EdU incorporation, Figure 2G) and migration (Figure 2H). These results clearly show that circNT5E silencing by targeted shRNA potently inhibited NSCLC cell growth, proliferation and migration.

### circNT5E silencing induces apoptosis activation in NSCLC cells

**Figure 3:**
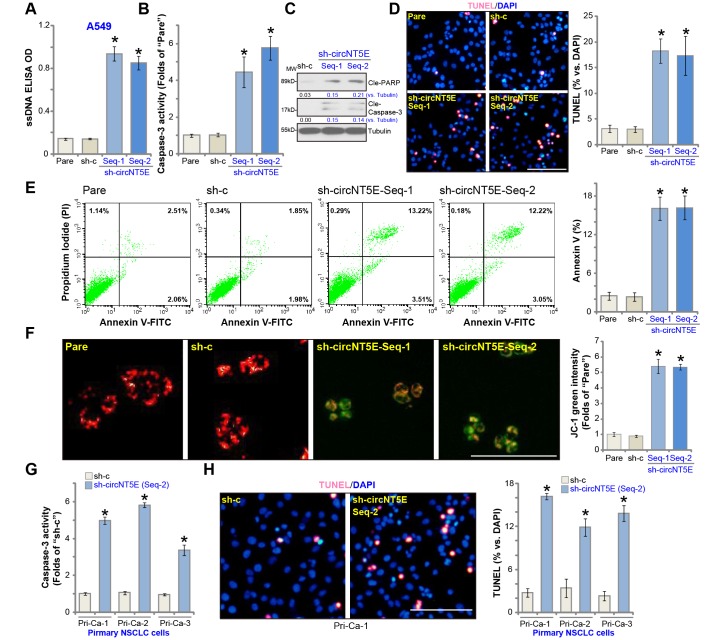
**circNT5E silencing induces apoptosis activation in NSCLC cells.** The stable A549 cells (**A**–**F**) or the primary human NSCLC cells (Pri-Ca-1/-2/-3, **G** and **H**) with the lentivirus-packaged circNT5E shRNA (“sh-circNT5E-Seq-1/2”) or the non-sense control shRNA (“sh-c”) were cultured for 48h, single strand DNA (ssDNA) contents (**A**), the relative caspase-3 activity (**B** and **G**) and cell apoptosis (**D**, **E** and **H**) were examined by the mentioned assays, with mitochondrial depolarization tested by JC-1 staining (**F**). Expression of listed apoptosis-associated proteins were tested, quantified and normalized to the loading control (**C**). Error bars stand for mean ± standard deviation (SD, n=5). * *P* <0.05 vs. “Pare”/“sh-c” cells. Experiments in this figure were repeated five times, and similar results were obtained. Bar= 100 μm (**D**, **F** and **H**).

Next the experiments were carried out to test whether circNT5E silencing could induce apoptosis activation in NSCLC cells. As shown in sh-circNT5E-expressing A549 cells (see Figure 2) the single strand DNA (ssDNA) contents, the characteristic marker of cell apoptosis, were significantly increased (Figure 3A). Further experimental results show that circNT5E shRNA resulted in a significant caspase-3 activity increase (Figure 3B) as well as cleavages of poly (ADP-ribose) polymerase (PARP) and caspase-3 (Figure 3C) in A549 cells, further indicating cell apoptosis activation. Furthermore, the stable A549 cells with the applied circNT5E shRNA showed increased nuclear TUNEL staining (Figure 3D) as well as the Annexin V staining (FACS results, Figure 3E). Following mitochondrial depolarization in apoptotic cells, JC-1 red fluorescence shall aggregate to form green monomers [32]. Indeed, significant JC-1 green fluorescence accumulation was detected in the circNT5E-silenced cells (Figure 3F). These results clearly show that circNT5E silencing induced apoptosis activation in A549 cells. In the primary human NSCLC cells (Pri-Ca-1/-2/-3), circNT5E silencing by sh-circNT5E-Seq-2 (see Figure 2) significantly increased the caspase-3 activity (Figure 3G) and nuclear TUNEL staining (Figure 3H), indicating apoptosis activation.

### Ectopic overexpression of circNT5E promotes NSCLC cell growth, proliferation and migration

**Figure 4:**
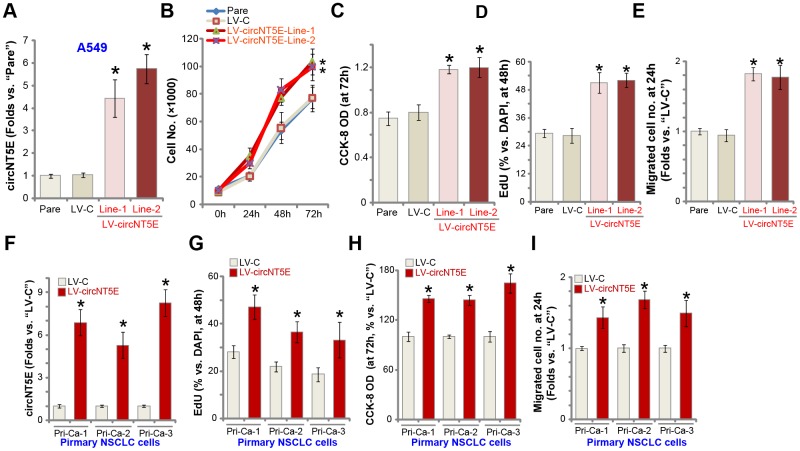
**Ectopic overexpression of circNT5E promotes NSCLC cell growth, proliferation and migration.** Stable A549 cells (**A**–**E**) or primary human NSCLC cells (Pri-Ca-1/-2/-3, **F**–**I**) with the lentiviral circNT5E expression construct (LV-circNT5E-Line-1/2, two lines) or the empty vector (LV-C) were cultured for applied time periods, circNT5E expression was tested by qPCR assays (**A** and **F**), cell growth (**B**), proliferation (**C**, **D**, **G** and **H**) and migration (**E** and **I**) were tested by the assays mentioned in the text. Error bars stand for mean ± standard deviation (SD, n=5). * *P* <0.05 vs. “Pare”/“LV-C” cells. Experiments in this figure were repeated five times, and similar results were obtained.

Next we tested whether ectopic overexpression of circNT5E could further promote NSCLC cell progression. A lentiviral circNT5E expression construct, LV-circNT5E, was transduced to A549 cells. Following selection two stable cell lines, LV-circNT5E-Line-1/2, were established, with mature circNT5E levels significantly increased (4-7 folds of control cells, Figure 4A). NT5E protein expression was unchanged with circNT5E overexpression in A549 cells (Supplementary Figure 1C). As shown, LV-circNT5E A549 cells grew significantly faster than the control cells with empty vector (LV-C) (Figure 4B). Additional experiments showed that overexpression of circNT5E promoted A549 cell growth, increasing the CCK-8 OD (Figure 4C) and BrdU incorporation (Figure 4D). Furthermore, “Transwell” assay results, Figure 4E, demonstrated that circNT5E overexpression significantly promoted A549 cell migration *in vitro*. In the primary human NSCLC cells, Pri-Ca-1/-2/-3, LV-circNT5E similarly increased circNT5E expression (Figure 4F), resulting in increases in BrdU incorporation (Figure 4G), CCK-8 OD (Figure 4H), and cell migration (Figure 4I). Collectively, these results show that ectopic overexpression of circNT5E promoted NSCLC cell growth, proliferation and migration.

### miR-134 inhibition attenuates circNT5E silencing-induced anti-NSCLC cell activity

**Figure 5:**
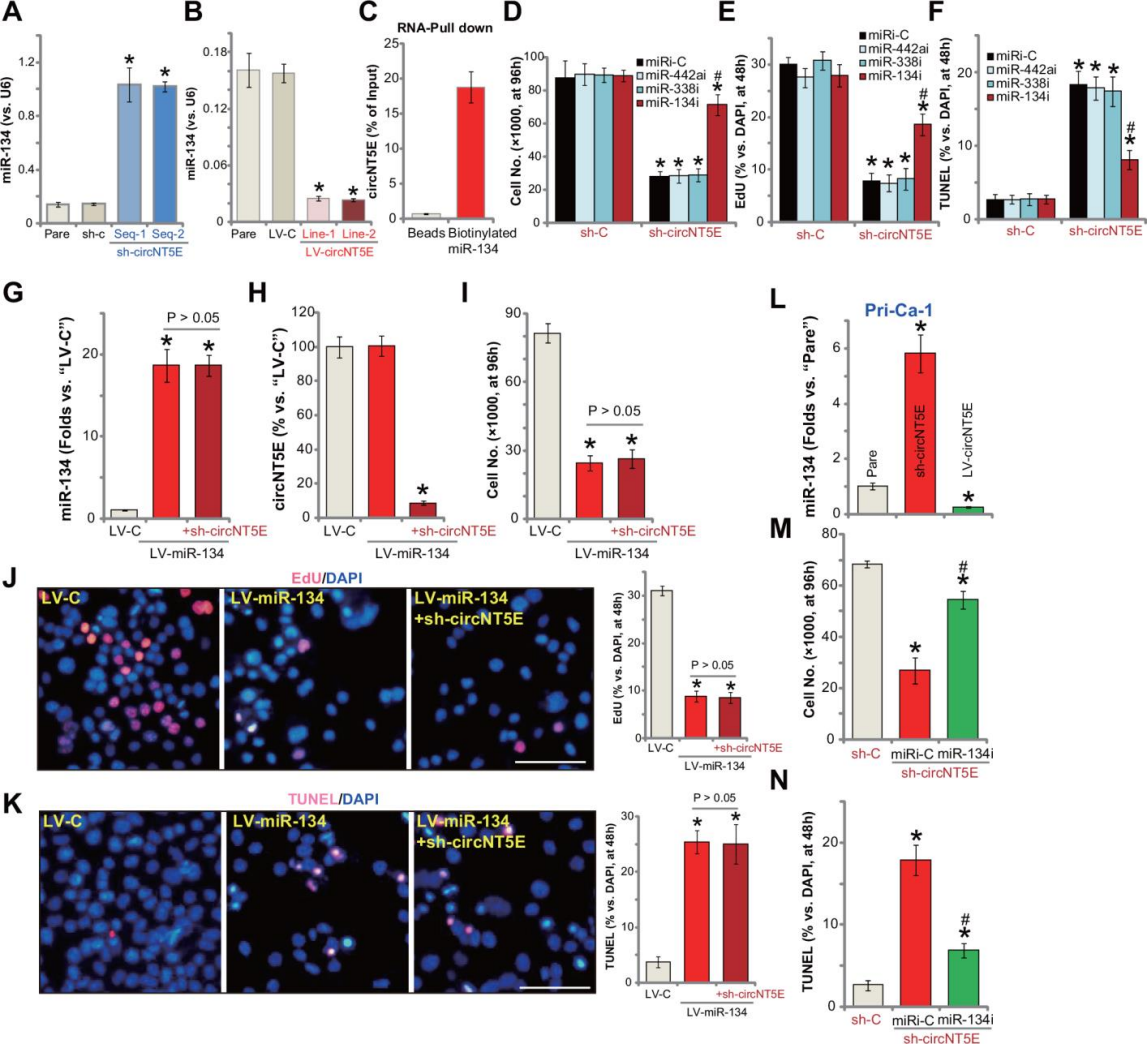
**miR-134 inhibition attenuates circNT5E silencing-induced anti-NSCLC cell activity.** Expression of mature miR-134 in stable A549 cells with the lentivirus-packaged circNT5E shRNA (“sh-circNT5E-Seq-1/2”), the non-sense control shRNA (“sh-c”) as well as in A549 cells with the lentivirus-packaged circNT5E expression construct (LV-circNT5E-Line-1/2, two lines) or the empty vector (LV-C) was shown (**A** and **B**); RNA-Pull down assay confirmed the direct association between biotinylated-miR-134 and circNT6E in A549 cells (**C**). Stable A549 cells with the lentivirus-packaged circNT5E shRNA (“sh-circNT5E-Seq-2”) or sh-c were further transfected with the applied microRNA inhibitors (500 nM, 48h), cells were cultured for applied time periods, cell growth, proliferation and apoptosis were tested by cell counting (**D**), EdU incorporation (**E**) and TUNEL staining (**F**) assays, respectively. A549 cells with the pre-miR-134 expression construct (LV-miR-134) were further infected with/without lentivirus-packaged circNT5E shRNA (“sh-circNT5E-Seq-2”), control cells were transduced with lentiviral empty vector (“LV-C”); Cells were further cultured for applied time periods, mature miR-134 and circNT5E expression was tested by qPCR assays (**G** and **H**); Cell growth (**I**), proliferation (**J**) and apoptosis (**K**) were tested. Stable primary human NSCLC cells, Pri-Can-1, with the lentivirus-packaged circNT5E expression construct (LV-circNT5E) or the lentivirus-packaged circNT5E shRNA (“sh-circNT5E-Seq-2”) were cultured, mature miR-134 expression was tested (**L**). Stable Pri-Can-1 cells with circNT5E shRNA were further transfected with miR-134 inhibitor (500 nM, 48h), cells were further cultured for applied time periods, cell growth (**M**) and apoptosis (**N**) were tested. Error bars stand for mean ± standard deviation (SD, n=5). “miRi” stands for non-sense control miRNA inhibitor. * *P* <0.05 vs. “sh-C”/“Pare”/“LV-C” cells. ^#^ *P* <0.05 vs. “miRi-C” cells. Experiments in this figure were repeated five times, and similar results were obtained. Bar= 100 μm (**J** and **K**).

Recent studies tested an important role of miR-134 in regulating NSCLC cell behaviors [28–30, 33]. It is mainly considered as a tumor-suppressive miRNA [28–30]. Although other studies also proposed the potential tumor-promoting activity of miR-134 [33]. The study by Wang et al., has shown that miR-134 can be directly sponged by circNT5E [24]. Here in A549 cells miR-134 levels increased over 5-6 folds following shRNA-mediated silencing of circNT5E (Figure 5A). On the contrary, in circNT5E-overexpressed A549 cells (see Figure 4) miR-134 levels were dramatically decreased (Figure 5B). These results indicate that miR-134 could be a circNT5E-targeting miRNA in A549 cells. To further support our hypothesis, the RNA-Pull down assay was performed. As shown, in A549 cells, circNT6E directly associated with biotinylated-miR-134 (Figure 5C). As the negatively control, the streptavidin-coated magnetic beads failed to bind to circNT6E (Figure 5C). These results suggest a direct binding between circNT6E and miR-134 in A549 cells.

It is possible that circNT5E silencing-induced anti-NSCLC cell activity is due to accumulation the tumor-suppressive miR-134. Here we show that transfection of miR-134 inhibitor (miR-134i) largely inhibited circNT5E shRNA (by sh-circNT5E-Seq-2)-induced inhibitions on A549 cell growth (Figure 5D) and proliferation (EdU incorporation, Figure 5E). Furthermore, circNT5E shRNA induced apoptosis activation, reflected by an increase in nuclear TUNEL staining, was also significantly inhibited by miR-134i (Figure 5F). Importantly, inhibition of other circNT5E’s potential targets, including miR-338 and miR-442 [24], was ineffective on circNT5E shRNA-induced inhibition on A549 cells (Figure 5D–5F).

We further propose that forced overexpression of miR-134 should mimic circNT5E shRNA-induced actions in NSCLC cells. Therefore, a lentiviral construct with pre-miR-134 (LV-miR-134) was transduced to A549 cells. Selected by puromycin stable cells were established. qPCR assay results, Figure 5G, confirmed overexpression of the mature miR-134 in LV-miR-134-expressing A549 cells (16-18 folds of control level). LV-miR-134 did not alter circNT5E expression in A549 cells (Figure 5H), but significantly inhibited cell growth (Figure 5I) and proliferation (Figure 5J), while simultaneously inducing significant apoptosis activation (Figure 5K). Importantly, in miR-134-overexpressed A549 cells, silencing circNT5E by sh-circNT5E-Seq-2 (Figure 5H), failed to further change cell functions, including growth, migration and apoptosis (Figure 5I–5K). Therefore, forced miR-134 overexpression not only mimicked, but also nullified, circNT5E shRNA-induced actions in NSCLC cells. In the primary human NSCLC cells (Pri-Ca-1), miR-134 levels were significantly increased following circNT5E shRNA, but was reduced with circNT5E overexpression (Figure 5L). Additionally, miR-134i inhibited circNT5E shRNA-induced growth inhibition (Figure 5M) and apoptosis activation (Figure 5N) in Pri-Ca-1 cells. Collectively, these results show that miR-134 accumulation mediated circNT5E silencing-induced anti-NSCLC cell activity.

## DISCUSSION

circRNAs are a large family of conserved noncoding RNAs (ncRNAs), generated from a non-canonical back splicing process, from a covalent bond between 5′ and 3′ ends of a single-stranded RNA [13, 16]. Recent studies have proposed the pivotal functions of circRNAs in NSCLC progression [11]. Unlike other ncRNAs, circRNAs can form highly stable circular structure via joining of the 3′ and 5′ terminals [10, 12, 13]. circRNAs function as sponges of its target miRNAs, regulating gene expression in cancer cells [10, 13, 14]. Dysregulation of circRNAs has been detected in NSCLC, essential for cancer development, progression and treatment-resistance [16].

Our results suggest that circNT5E is possibly an oncogenic circRNA in NSCLC. Its expression is significantly elevated in human NSCLC tissues and in established/primary human NSCLC cells, but low in lung epithelial tissues and cells. In A549 and primary human NSCLC cells, circNT5E silencing, by targeted shRNA, potently inhibited cell growth, proliferation and migration, whiling inducing apoptosis activation. Conversely, exogenous overexpression of circNT5E, by a lentiviral construct, promoted A549 cell growth, proliferation and migration. These results suggest that circNT5E could be an important and novel therapeutic target of NSCLC.

MiRNAs, dysregulated in human cancer cells [34], are small ncRNAs binding to the 3’-UTR of target genes [34–36]. Recent studies have indicated that miR-134 exerts tumor-suppressive functions in NSCLC and other cancers [28–30]. Sun et al., reported that by targeting cyclin D1, miR-134 inhibited NSCLC cell proliferation, migration, invasion, and promoting apoptosis [28]. Li et al., have shown that miR-134 expression negatively correlated with invasive potential and epithelial to mesenchymal transition (EMT) phenotype of NSCLC cells. Furthermore, miR-134 suppressed EMT in NSCLC cells by directly targeting Forkhead Box M1 (FOXM1) [30]. Additionally, the study by Qin et al., demonstrated that miR-134 targeted and downregulated epidermal growth factor receptor (EGFR), inhibiting NSCLC cell growth [29].

circNT5E can sponge miR-134 and possible other tumor suppressive miRNAs [24]. The results of the current study indicate that miR-134 is the primary target of circNT5E in NSCLC cells. First, the RNA-Pull down assay in A549 cells confirmed the direct association between biotinylated-miR-134 and circNT6E. miR-134 levels were significantly increased in circNT5E-silenced A549 cells, but downregulated with circNT5E overexpression. Importantly, exogenous overexpression of miR-134 by a lentiviral construct potently inhibited A549 cell progression *in vitro*, mimicking circNT5E shRNA-induced activity. Conversely, miR-134 inhibition, via transfection of its inhibitor, largely attenuated circNT5E shRNA-induced anti-A549 cell activity. Furthermore, circNT5E shRNA was ineffective in the miR-134-overexpressed A549 cells. Importantly, in human NSCLC tissues and cells, circNT5E upregulation correlates with miR-134 downregulation. These results indicate that circNT5E promotes NSCLC cell progression possibly by sponging miR-134. miR-134 should be the direct and main target of circNT5E in NSCLC cells.

The other important miRNA that can be sponged by circNT5E is miR-422a, a tumor-suppressive miRNA [24, 37–41]. In human glioblastoma cells, miR-422a inhibits PI3KCA expression, Akt activation and cancer cell growth [40]. In head and neck squamous cell carcinoma, miR-422a promotes loco-regional recurrence [41]. LncRNA 00858 promotes NSCLC cell progression by sponging miR-422a [39]. In this study, we show that although miR-422a levels were increased following circNT5E shRNA (data not shown), miR-422a inhibition failed to change circNT5E-induced cytotoxicity in A549 cells. Furthermore, forced overexpression miR-422a, by transfection of its mimic, showed no significant effect on A549 cell growth and proliferation (data not shown). Therefore, miR-422a is unlikely a primary target of circNT5E in mediating its function in NSCLC cells. Future studies will be needed to further explore the potential function and underlying mechanisms of circNT5E-miR-134 axis in NSCLC cell growth *in vivo*, for example using mice xenografts.

## CONCLUSION

Collectively, we conclude that circNT5E, elevated in NSCLC tissues and cells, promotes human NSCLC cell progression possibly by sponging miR-134. circNT5E-miR-134 axis could be a novel therapeutic target of NSCLC.

## MATERIALS AND METHODS

### Chemicals and reagents

The antibodies utilized in this study were purchased from Abcam (Cambridge, MA). The reagents for cell culture, including DMEM, fetal bovine serum (FBS) and antibiotics, were purchased from Hyclone (Logan, UT). Puromycin, polybrene and all other chemicals were provided by Sigma-Aldrich (St. Louis, Mo). All the primers, sequences, virus and expression constructs were designed, provided and verified by Shanghai Genechem Co. (Shanghai, China).

### Culture of established cell lines

The established A549 NSCLC cells and the BEAS-2B lung epithelial cells were provided by Dr. Jiang [25]. Cells were cultured in DMEM with 10% FBS and 1% penicillin/streptomycin [25]. Cells were routinely subjected to mycoplasma/microbial contamination examination every 3-6 months. STR profiling, population doubling time, and morphology were always checked to verify the genotype.

### Primary human cells and tissues

In this study the primary human NSCLC cells were provided by Dr. Jiang [25, 42]. The primary cells were derived from three written-informed consent NSCLC patients (male, 53 to 59-year old, stage-III), named as “Pri-1/-2/-3”. Additionally, the primary human lung epithelial cells, derived from lung epithelial tissues, were provided by Dr. Jiang as well [25, 42]. Primary human cells were cultured in complete RPMI medium with necessary supplements and antibiotics [25, 42]. Five (5) written-informed consent primary NSCLC patients (male, 46 to 64-year old, stage-III) were enrolled. Patients received no prior treatment before surgery. The lung cancer tissues and paired paracancer lung epithelial tissues were obtained at the time of surgery, stored in liquid nitrogen and subjected to further biomedical analyses. The protocols of this study were approved by the Ethics Committee of authors institutions, in according to Declaration of Helsinki.

### Quantitative real-time PCR (qPCR)

Total cellular and tissue RNAs were isolated by the Trizol reagent (Thermo-Fisher Invitrogen, Grand Island, NY), quantified and reverse transcribed as described [43]. qPCR was carried out using the previously-described protocol, using U6 small nuclear RNA as the internal control [24]. All the primers were reported early [24].

### Western blotting

The equivalent amounts of total cellular lysates (40 μg per treatment) were separated by 10-12% of SDS-PAGE gels, then transferred to the polyvinylidene fluoride (PVDF) blots (Merck Millipore, Darmstadt, Germany). After blocking in 10% non-fat milk, the blots were incubated with the applied primary antibodies, followed by incubation with corresponding secondary antibodies. Antibody-antigen binding was detected by an enhanced chemiluminescence (ECL) substrate kit (Thermo-Fisher Invitrogen), with the results quantified by an ImageJ software (NIH, Bethesda, MD).

### Cell Counting Kit-8 (CCK-8)

Cells were initially seeded into 96-well plates at 5×10^3^ cells per well. Following incubation for 72h, CCK-8 solution (10 μL/well, Dojindo Molecular Technologies, Gaithersburg, MD) was added to each well. After incubation for another 3h, CCK-8 optical density (OD) values were measured at test-wavelength of 450 nm.

### Colony formation

A549 and primary NSCLC cells were initially seeded at 1×10^4^ cells per 10-cm dish. Colony formation assays were conducted at day-10, with the colonies fixed and stained (with 1% crystal violet solution). The number of colonies was counted manually.

### *In vitro* migration

A549 and primary NSCLC cells (1 × 10^5^ cells in 300 μL serum-free medium) were seeded into the upper part of each “Transwell” chambers (12-μm pore size, BD Biosciences, Heidelberg, Germany). The lower compartments were filled with medium with 10% FBS. Following incubation for 24h, non-migrated cells on the upper surface were wiped out. The migrated cells, on the lower surface, were fixed and stained.

### EdU assay of proliferation and cell counting assay

NSCLC cells were seeded into six-well plates at 8×10^4^ cells per well, and cultured for 48h. An EdU (5-ethynyl-20-deoxyuridine) Apollo-567 Kit (RiboBio, Guangzhou, China) was applied. EdU and DAPI dyes were added to NSCLC cells for additional 4h. Under a fluorescent microscope cell nuclei were visualized. For each condition total 800 nuclei in five random views were included to calculate the EdU ratio (EdU/DAPI×100%). For cell counting assay 1×10^4^ NSCLC cells (with applied genetic treatments) per well were initially seeded (at 0h), cells were cultured for applied time periods with cell number recorded.

### Annexin V FACS

Following the applied genetic treatments, Annexin V-FITC and Propidium Iodide (PI) dyes (each at 10 μg/mL, BD Pharmingen, San Diego, CA) were added for 30 min under the dark at room temperature. Cell apoptosis was analyzed by a flow cytometry machine (Beckman Coulter, Brea, CA).

### Caspase-3 activity assay

In A549 and primary human NSCLC cells with the applied genetic treatments, the caspase-3 activity was tested using the previously-described protocol [44].

### ssDNA ELISA

In apoptotic cells, single strand DNA (ssDNA) will be accumulated. ssDNA contents were measured from 30 μg cell lysates per treatment, using a commercial ssDNA ELISA kit (Roche, Basel, Switzerland), with the ssDNA ELISA absorbance measured at 405 nm.

### circNT5E shRNA

Two shRNAs targeting non-overlapping sequences (“Seq-1/2”) of circNT5E [24] were individually sub-cloned into GV248 (hU6-MCS-Ubiquitin-EGFP-IRES-puromycin) construct (Shanghai Genechem Co.), then transfected to HEK-293 cells with lentivirus package plasmid mix (Shanghai Genechem Co.). The generated circNT5E shRNA lentivirus (“LV-circNT5E shRNA”) was added to cultured NSCLC cells (in polybrene medium). Following selection by puromycin (5.0 μg/mL, for 4-5 passages), stable cells were established. Silencing of circNT5E (over 90% knockdown efficiency) in stable cells was confirmed by qPCR. Control cells were transfected with lentiviral scramble control shRNA.

### Ectopic overexpression of circNT5E or miR-134

The full-length pre-circNT5E [24] and pre-miR-134 were synthesized by Shanghai Genechem Co, sub-cloned to a lentiviral GV248 construct (Shanghai Genechem Co.). The construct, together with lentivirus package plasmid mix, were co-transfected to HEK-293 cells [45] to generate pre-circNT5E-expressing lentivirus (“LV-circNT5E”) and pre-miR-134-expressing lentivirus (“LV-miR-134”). Following filtration and enrichment, LV-circNT5E or LV-miR-134 was added to cultured NSCLC cells. Puromycin was added to select stable cells. Control cells were infected with lentivirus with empty vector. Expression of mature circNT5E or miR-134 in the stable cells was always tested.

### JC-1 assaying of mitochondrial depolarization

JC-1 aggregating in cell mitochondria is a characteristic marker in apoptotic cells, forming green monomers [32]. NSCLC cells with applied genetic treatments were seeded into 12-well plates (3 × 10^4^ cells in each well), stained with JC-1 (5 μg/mL) and tested by a fluorescence spectrofluorometer at 550 nm. The representative JC-1 images, merging the green fluorescence image (at 550 nm) and the red fluorescence image (at 650 nm), were also presented.

### Transfection of miR mimic and miR inhibitors

A549 and primary human NSCLC cells were seeded initially into the six-well plates at 50% confluence. Cells were transfected with 500 nM of the applied miR inhibitor or miR inhibitor control (purchased from Shanghai Genechem Co.) by Lipofectamine 2000 (Thermo-Fisher Invitrogen) for 48h. Afterwards, expression of targeted miRNA was tested by qPCR.

### RNA-Pull down assay

Using a previously-described protocol [24, 46], the capture of miR-134-bound ceRNAs in a pull-down assay with biotinylated miR-134 was carried out (Pierce Magnetic RNA-Protein Pull-Down Kit, Pierce Biotechnology). In brief, A549 cells were transfected with biotinylated miR-134 mimic or control mimic (100 nmol/L) for 48h, and cells were harvested using the described lysis buffer [46]. The biotin-captured RNA complex was pulled down by incubating the cell lysates with the streptavidin-coated magnetic beads [24]. The bound RNA was purified using an RNeasy Mini Kit (QIAGEN), with expression of the ceRNA, circNT6E, in the bound fractions tested by qPCR. circNT6E levels were normalized to the input controls.

### FISH

As described early [24], FITC-labeled circNT5E was designed and synthesized by Shanghai Genechem Biotech (Shanghai, China). A fluorescent in situ hybridization kit (RiboBio, Guangzhou, China) was utilized to test the probe signals. The cells with no FITC-labeled circNT5E were control cells. The fluorescent was detected under a fluorescence microscope (Leica, Shanghai, China).

### Statistical analysis

Statistical analyses were performed by SPSS 21.0 software (SPSS Inc., Chicago, IL). The t-test or the One-Way Analysis of Variance (ANOVA) was applied to measure variables among groups. All data are presented as mean ± standard deviation (SD). P values < 0.05 were considered statistically significant.

## Supplementary Material

Supplementary Figure 1
