# Supplementary material for: The circular RNA NT5E promotes non-small cell lung cancer cell growth via sponging microRNA-134
Source: Aging (Albany NY). 2020 Feb 25;12(4):3936–49. doi: 10.18632/aging.102861 (PMC7066882; doi:10.18632/aging.102861)
Supplement: Supplementary Figure 1 [file aging-12-102861-s001..pdf]

SUPPLEMENTARY MATERIALS

Supplementary Figure

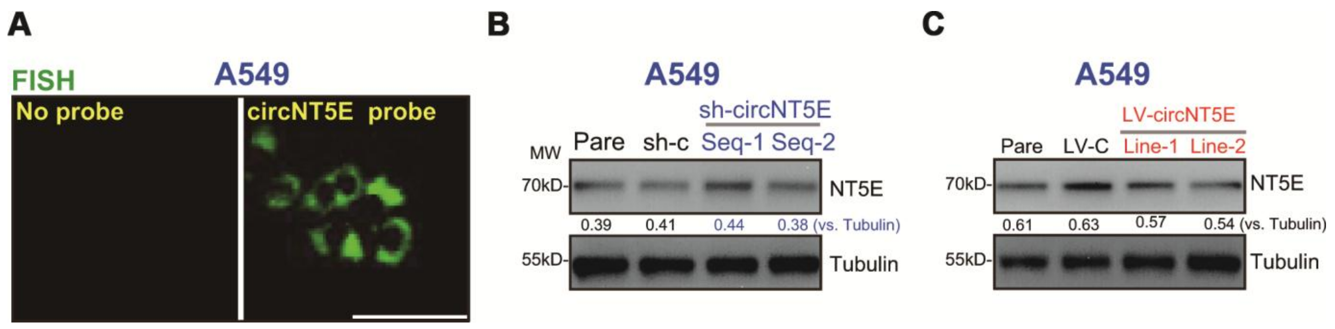

Supplementary Figure 1. FISH showed the localization of circNT5E in A549 cells. (**A**). Expression of NT5E and Tubulin (the loading control) in stable A549 cells with circNT5E shRNA (“sh-circNT5E-Seq-1/2”), the non-sense control shRNA (“sh-c”), lentiviral circNT5E expression construct (“LV-circNT5E-Line-1/2”) or the empty vector (“LV-C”), was shown (**B** and **C**, results were quantified). Bar= 50 µm (**A**).
